# Morphological, radiographic, three-dimensional computed tomographic, and histological features of the primary upstroke and downstroke muscles and bones in the domestic duck (*Anas platyrhynchos domesticus*) and the cattle egret (*Bubulcus ibis, Linnaeus, 1758*), reflecting the evolutionary transition towards the irreversible flightlessness

**DOI:** 10.1186/s12917-023-03649-6

**Published:** 2023-08-25

**Authors:** Hanaa M. El-Ghazali, Ahmed Abdelbaset-Ismail, Nehal I. A. Goda, Mohamed Aref

**Affiliations:** 1 Anatomy and Embryology Department, Faculty of Veterinary Medicine, Zagazig University, Zagazig, 44519 El-Sharkia Egypt; 2 Department of Surgery, Anesthesiology, and Radiology, Faculty of Veterinary Medicine, Zagazig University, Zagazig, 44519 El-Sharkia Egypt; 3 Department of Histology and Cytology, Faculty of Veterinary Medicine, Zagazig University, Zagazig, 44519 El-Sharkia Egypt

**Keywords:** Domestic duck, Cattle egret, Domestication, Flightlessness, Anatomy, Radiography, Computed tomography, Histology

## Abstract

**Background:**

The purpose of this study was to explore whether domestication could lead to evolutionary changes towards flightlessness in the domestic duck *(Anas platyrhynchos domesticus)* compared to the cattle egret *(Bubulcus ibis)* as a nonflying and flying biological model, respectively. Bones of the pectoral girdle (scapula, clavicle, and coracoid) and the foramen triosseum were comparatively assessed using anatomical, radiographic, and 3D computed tomographic (CT) studies. Additionally, the muscles *pectoralis* and the *supracoracoideus* were histologically and immunohistochemically assessed.

**Results:**

Among the differences observed, radiographically, the distance between the paired clavicles was significantly wider (*p* < 0.05) in the domestic duck (mean ± SD 1.43 ± 0.23 cm) compared with the cattle egret (0.96 ± 0.13 cm). Unlike cattle egrets, there was no connection between the sternum and the hypocladium of furcula in domestic ducks. The scapula, clavicle, coracoid, sternum, and humerus were considerably longer in domestic ducks than in cattle egrets. The foramen triosseum appeared significantly (*p* < 0.01) wider in domestic ducks (0.7 ± 1.17 cm) compared to cattle egrets (0.49 ± 0.03 cm). Histologically, compared to cattle egrets, the muscle fibers in domestic ducks were loosely connected and contained fewer nuclei and perimysial/endomysial spaces. A higher myoglobin expression was evident in cattle egrets compared with domestic ducks.

**Conclusions:**

Results of this study indicate that the bones and muscles of the pectoral girdle generally show specific morphological and structural changes reflective of the loss of prerequisites associated with flight behavior in domestic ducks due to domestication effects compared to cattle egrets.

## Background

Birds can be classified according to their flying ability into two broad categories, non-flying (flightless) and flying birds [1]. Flightless birds have lost, through evolution, the ability to fly. There are two main differences between non-flying and flying birds, including poorly developed wing bones and the absent or significantly reduced carina (keel) of the sternum (breastbone) [2]. The cattle egret (*Bubulcus ibis,* Linnaeus, 1758) is a universal species of herons belonging to the Ardeidae family and commonly lives in warm countries in agricultural areas and near the water [3]. In Egypt, it is also called “Abu-Qerdan” or “Farmer's Friend” [4]. They are often seen in the fields on the backs of cattle, buffaloes, or other large animals, hunting ticks and flies [3].

Of the non-flying birds, despite their ancestral species, many domesticated birds including the domestic duck (*Anas platyrhynchos domesticus*) have lost the ability to fly for extended periods [5]. As reported, the adaptation of birds to the occasional lifestyle could lead to some morphological changes in their musculoskeletal system [6].

In birds, the pectoral girdle and associated muscles including the *pectoralis* and *supracoracoideus* are the primary apparatus used to operate the wings’ downstroke and upstroke during flying [7]. Functionally, the pectoral girdle allows the body to withstand the movements of wing strokes and transfer force to the sternum and provides an extra anchoring surface for the flight muscles [8]. Moreover, it also provides wings with the strength and range of motion during flight [9]. Anatomically, the pectoral girdle of birds consists of the scapula, clavicle, and coracoid. These three bones are articulated together in the nearby epiphyses to form the so-called *foramen triosseum,* where the tendons of flight muscles, the *pectoralis*, and *supracoracoideus* muscles communicate [10]. The *pectoralis* and *supracoracoideus* are the primary downstroke (depressor) and upstroke (elevator) muscles, respectively, of the wings during the flight of birds. The *supracoracoideus* is located deep in the *pectoralis* (pars thoracicus), and its highly oriented fascicles originate from the dorsal half of the keel and the coracoclavicular membrane. Through the craniodorsal surface of the triosseal canal, its tendon of insertion passes and ultimately inserts onto the dorsoproximal aspect of the humerus [8]. The *pectoralis* is a paired muscle located on the ventral surface of the chest area. It arises mainly from the lateral and caudal surfaces of the breastbone. In some avian species, it is divided into two distinct parts: *pars sternobrachialis* and *pars thoracobrachialis*. The aponeuroses of these two parts join laterally and insert onto the ventral surface of the humerus [11]. Histologically, the structure of both muscles varies based on the avian flight capacity. Comparable to domestic chickens (*Gallus domesticus*), the pectoral and *supracoracoideus* muscles in the pigeon (*Columba livia*) showed microscopically abundant myofibers and nuclei as well as wider perimysial spaces separating the muscle fascicles [11]. In turkey *(Meleagris gallopavo)* muscles, the myofibers are surrounded by a thin collagen layer of the endomysium [12]. In ducks (*Anseriformes*) and pigeons (*columbiformis*), the nuclei appeared elongated, whereas in chickens (*galliformis*) appeared oval and peripherally located under sarcolemma [13, 14]. Despite there are several studies have evaluated the avian flight muscles [11, 15, 16], however, to the best of the authors’ knowledge, there is no information regarding the comparative evaluation of the flight muscles and bones in the domestic duck (*Anas platyrhynchos domesticus*) and the cattle egret (*Bubulcus ibis*).

Thus, the main purpose of this study was to describe, for the first time, the anatomic, radiographic, computed tomographic, and histological features of the primary upstroke and downstroke muscles and bones in the domestic ducks (*Anas platyrhynchos domesticus*) comparably to the cattle egret (*Bubulcus ibis*).

### Materials and Methods

#### Birds

A total of 20 clinically healthy adult birds of both sexes [the domestic duck (*Anas platyrhynchos domesticus*, *n* = 10) and the cattle egret (*Bubulcus ibis*, *n* = 10)] were involved in this study. Ducks were purchased at private local markets while cattle egrets were collected from the field in the nearby villages in Sharkia governorate, Egypt. All the birds were humanely transported in a ventilated closet to the Faculty of Veterinary Medicine, Zagazig University. Birds were housed in suitable cages under specific conditions of controlled temperature and humidity. Food and water were available ad libitum. Birds were allowed to acclimatize for two weeks before starting the actual experiment. All methods were performed in accordance with the relevant guidelines and regulations of Zagazig University, Egypt with the approval of the Institutional Animal Care and Use Committee (IACUC approval # ZU-IACUC/2/F/234/2022) of Zagazig University. For these birds, the anesthesia was initially induced using a confined box provided with halothane gas (1–3 mL on a cotton ball per bird), and euthanasia was then immediately obtained by decapitation using sharp blades with complete loss of consciousness within 20 s after removing the head and outflow of the cerebral spinal fluid (CSF) from the severed spinal cord, as described previously [17, 18]. All methods are reported in accordance with ARRIVE guidelines [19].

### Radiographic examination

Immediately after euthanasia, plain ventrodorsal radiographic imaging was conducted to detect the appearance, structures, and position of the pectoral girdle as well as foramen triosseum using a conventional Toshiba Rotanode x-ray machine (POX-300BT, Japan) with standard radiographic parameters (50 kv, and 5 mAs) and object-to-focal distance as described previously for the Peking domestic duck [20].

### Three–dimensional computed tomographic (3D CT) imaging

Birds (three from each species) were examined using a computed tomography (CT) imaging system (1.5-T helical CT scanner, GE, Hi-speed NX\I Dual slice CT, Japan) as previously described in Peking ducks (*Anas platyrhynchos var. domestica*) [21]. Each bird was ventrodorsally (supine) or dorsoventrally (prone) positioned on the CT table. The ventrodorsal, ventrolateral, dorsoventral, and dorsolateral projections were performed to examine the pectoral girdle bones and foramen triosseum in both species.

### Anatomical and morphometric analysis

We performed two measurements to determine the body size: the body length (was measured from the tip of the beak to the end of the rump using a graduated tape) and body weight (was obtained using a digital scale). Immediately after euthanasia, the birds were skinned and eviscerated. Afterward, the specimens (pectoral girdle containing muscles and bones) were collected, washed, fixed in a mixture of 10% formalin, 3% glycerin, and 1% thymol, and then dissected. The muscles and ligaments related to the triosseum foramen were then grossly examined and identified. Bones of the pectoral girdle were carefully dissected and prepared as previously described [22]. The lengths of the bones forming the triosseum foramen and the pectoral girdle (scapula, coracoids, clavicle, and humerus), and the sternum were also measured. Also, the distance between the paired clavicles was measured using 3D CT scans of domestic ducks and cattle egrets. The anatomical nomenclature was made following Nomina Anatomica Avium [8] and Nomina Anatomica Veterinaria [23] whenever possible.

### Histology and immunohistochemistry

For histology [24], the specimens collected from M. *pectoralis* and M. *supracoracoideus* were fixed in 10% buffered neutral formalin and then dehydrated and cleared in xylene. All specimens were then embedded in wax paraffin and the obtained sections (5-μm thickness) were stained with a standard Harris’s hematoxylin and eosin staining to examine their general histological structure. For immunohistochemistry, the sections were deparaffinized by xylene, re-hydrated, and then washed with phosphate-buffered saline (PBS). After immersing in a 0.3% hydrogen peroxide solution, the sections were incubated in a 10% normal rabbit serum for blocking the nonspecific binding sites of immunoglobulins. After blocking, the sections were incubated with primary myoglobin polyclonal antibody (Cat. No. LS-B16910; clone aa1-154, Lifespan Biosciences, Inc., WA, USA). After washing, the sections were incubated with horseradish peroxidase (HRP)-conjugated secondary antibody. After incubation with the chromogen diaminobenzidine (DAB) for 2–4 min at room temperature, sections were washed, then counterstained with Mayer's hematoxylin, dehydrated, cleared, and then mounted with Canada balsam**.** All stained sections were examined by a standard light microscope (Olympus BX 21) and photographed by Canon PowerShot G10.

### Histomorphometric analysis

The histomorphometric analysis was applied on six representative fields per bird and analyzed using ImageJ software (Fiji ImageJ; NIH) for the quantitative measurements of 1) thickness of muscle bundles (um) of pectoral and supracoracoideus muscles of cattle egret and duck using H&E-stained photomicrographs at × 100 magnification, 2) area % of connective tissue within pectoral and supracoracoideus muscles of cattle egret and duck by using H&E-stained photomicrographs at × 100 magnification, and 3) the number of nuclei per defined area (400 um^2^) within pectoral and supracoracoideus muscles of cattle egret and duck by using H&E-stained photomicrographs at × 400 magnification.

### Statistical analysis

Descriptive analysis was performed, and the obtained data were displayed as mean ± SD. An unpaired 2-tailed t-test was employed to compare the means of all morphometric measurements in domestic ducks and cattle egrets. Data analysis was conducted with Prism GraphPad Software-version 8 (GraphPad Software Inc, California). For all analyses, statistically significant was set at a value of *p* < 0.05.

## Results

### Radiography and 3D computed tomography

Tables 1 and 2 show the statistically analyzed measurements of the triosseum foramen and bones of the pectoral girdle in domestic ducks and cattle egrets. The distance between the paired clavicles was significantly wider (*p* < 0.05) in domestic ducks (1.43 ± 0.23 cm) than that in cattle egrets (0.96 ± 0.13 cm). On 3D CT scan images, the structures of the pectoral girdle and triosseum foramen that were detected in a dorsolateral view (Fig. 2**)**, ventrodorsal and ventrolateral views (Fig. 5**)**, dorsal, dorsolateral, and ventral views (Fig. 8**)** were identified and labeled**.** On radiographs, the scapula, coracoid, clavicle, humerus, and triosseum foramen were identified and labeled (Fig. 3**)**.

**Table 1:** The different morphometric measurements of the domestic duck's body, length of some bones, and the width of the triosseum foramen in relation to body length

**Width of the triosseum foramen/cm**	**Clavicle length to body length / %**	**Clavicle length/cm**	**Humerus length to body length / %**	**Humerus length/cm**	**Coracoid length to body length / %**	**Coracoid length/cm**	**Scapula length to body length / %**	**Scapula length /cm**	**Sternum length to body length / %**	**Sternum length /cm**	**Body length** **/cm**	**Body weight** **/gm**	**Number**
0.7	12	6	24	12.5	13	7	19	9.7	25	13	52	1700	1
0.7	12	6.5	23	12.5	13	7	20	11	24	13.3	55	1650	2
0.7	13	7.3	23	13	13	7.5	20	11.3	23	13	57	2400	3
0.7	12	7.5	22.7	14.8	11	7.2	17	11	20	13.2	65	3500	4
0.7	11	7	20	13	11	7.3	16	10.5	22	14	65	3500	5
0.7	13	7.5	25	14	13	7.3	20	11.3	23	13	57	2250	6
0.7	13	7.4	23	13.5	12	7.1	19	11	23	13.5	58	2000	7
0.7	13	7.3	22	13	13	7.5	19	11.3	22	13	58	2250	8
0.7	12	7.4	24	15.1	12	7.4	17	11	22	14	63	3000	9
0.7	12	7.5	22	14	12	7.5	17	11.2	25	16	65	4500	10
0.7	12.3	7.14	22.87	13.54	12.3	7.28	18.4	10.93	22.9	13.6	59.5	2675	Mean
1.17	0.67	0.51	1.37	0.9	0.82	0.19	1.51	0.49	1.52	0.93	4.67	924.44	SD

**Table 2:** The different morphometric measurements of the cattle egret's body, length of some bones, and the width of the triosseum foramen in relation to body length

**Width of the triosseum foramen/cm**	**Clavicle length to body length / %**	**Clavicle length/cm**	**Humerus length to body length / %**	**Humerus length/ cm**	**Coracoid length to body length / %**	**Coracoid length/cm**	**Scapula length to body length/%**	**Scapula length / cm**	**Sternum length to body length / %**	**Sternum length /cm**	**Body length** **/cm**	**Body weight** **/gm**	**Number**
0.5	10	4.3	21	9	9	3.7	12	5	15	6.5	42	295	1
0.5	11	4.3	23	9	9	3.7	13	5	16	6.5	40	340	2
0.5	11	4.3	22	9	9	3.5	9	3.5	12	5	40.5	375	3
0.4	11	4.3	24	9	9	3.5	13	5	14	5.5	38	250	4
0.5	10	4.1	22	8.8	9	3.7	12	4.8	13	5.3	40.5	345	5
0.5	10	4.3	21	9	9	3.7	11	4.6	13	5.5	42	285	6
0.5	9	4.1	20	9	8	3.7	11	5	14	6.3	45	340	7
0.5	10	4.3	21	9	9	3.7	11	4.6	13	5.5	42	330	8
0.5	9	4	20	9	8	3.5	11	4.8	15	6.6	44	300	9
0.5	10	4.3	20	9	8	3.7	11	4.8	15	6.7	44.5	350	10
0.49	10.1	4.23	21.4	8.98	8.7	3.64	11.4	4.71	14	5.94	41.85	321	Mean
0.03	0.74	0.12	1.35	0.06	0.48	0.09	1.17	0.45	1.25	0.64	2.19	37.40	SD

### Gross anatomical findings

**Fig. 1:**
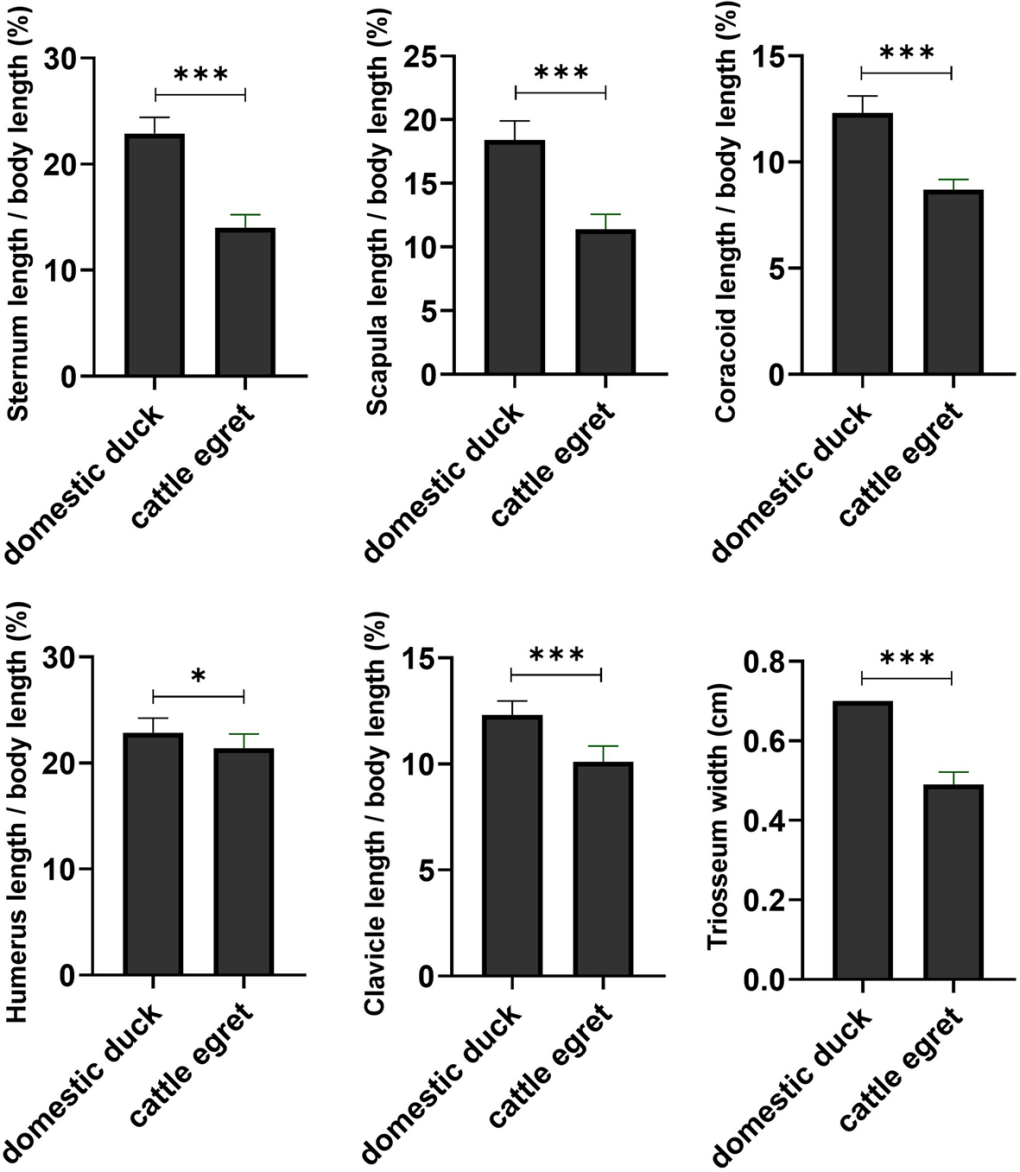
The statistical differences in the length of the sternum, scapula, coracoid, humerus, and clavicle in relation to the body length, as well as the width of triosseum foramen in domestic ducks compared to cattle egrets. *p = 0.03, ***p < 0.0001

**Fig. 2:**
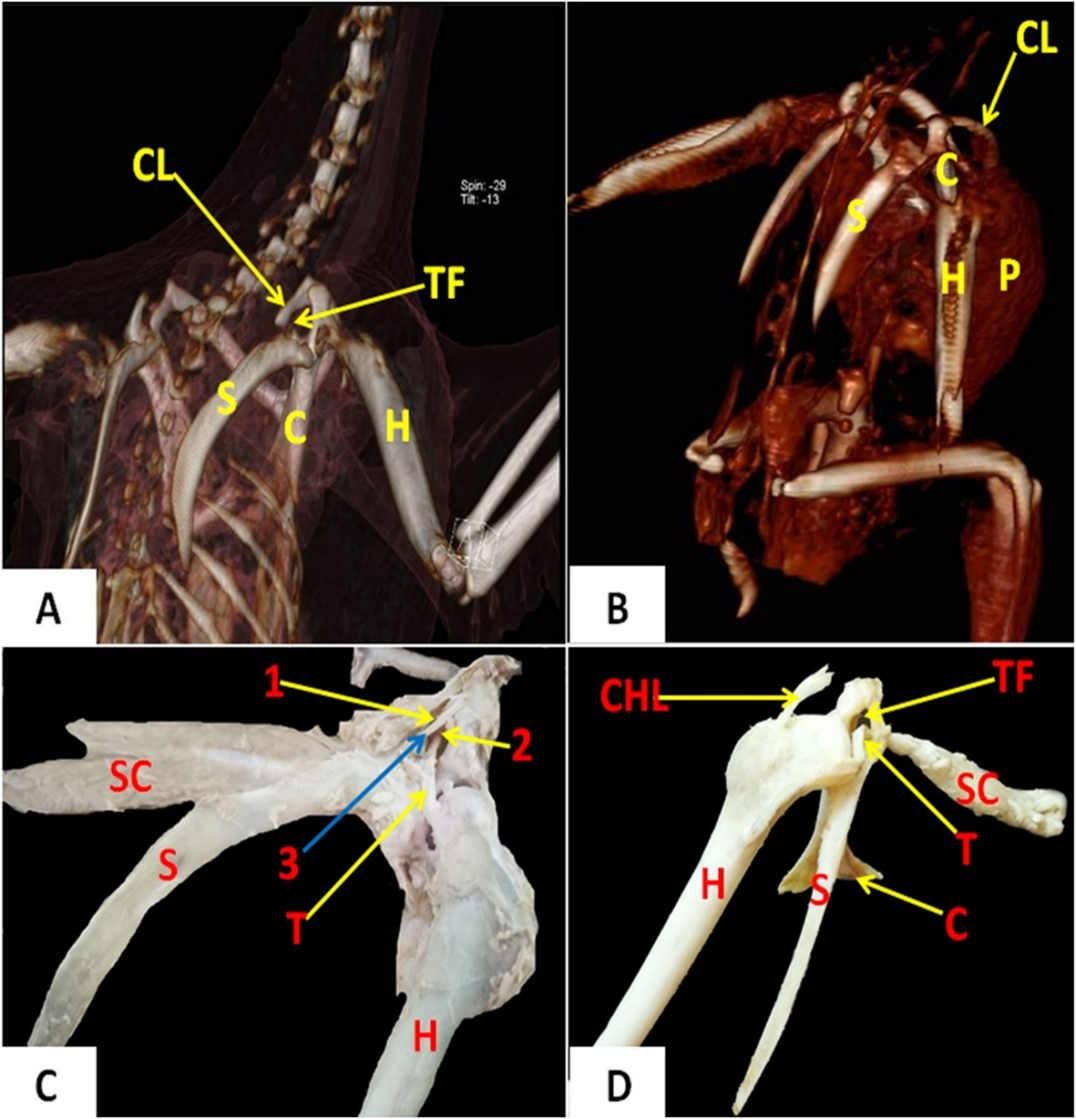
A representative dorsolateral 3D CT image of bones forming the pectoral girdle and triosseum foramen in domestic ducks (**A**) and cattle egrets (**B**). A representative photomacrograph of domestic ducks (**C**) and cattle egrets (**D**) (dorsolateral view) showing bones that formed the pectoral girdle and triosseum foramen. *S* Scapula, *C* Coracoid, *CL* Clavicle, *H* Humerus, *TF* Triosseum foramen, *P* Pectoral muscle, *SC* Supracoracoid muscle, *T* Tendon of Supracoracoid muscle, *CHL* Coracohumeral ligament. 1: Craniomedial part of triosseum foramen, 2: Caudolateral part of triosseum foramen, 3: Medial coracoclavicular and medial coracoscapular ligaments

**Fig. 3:**
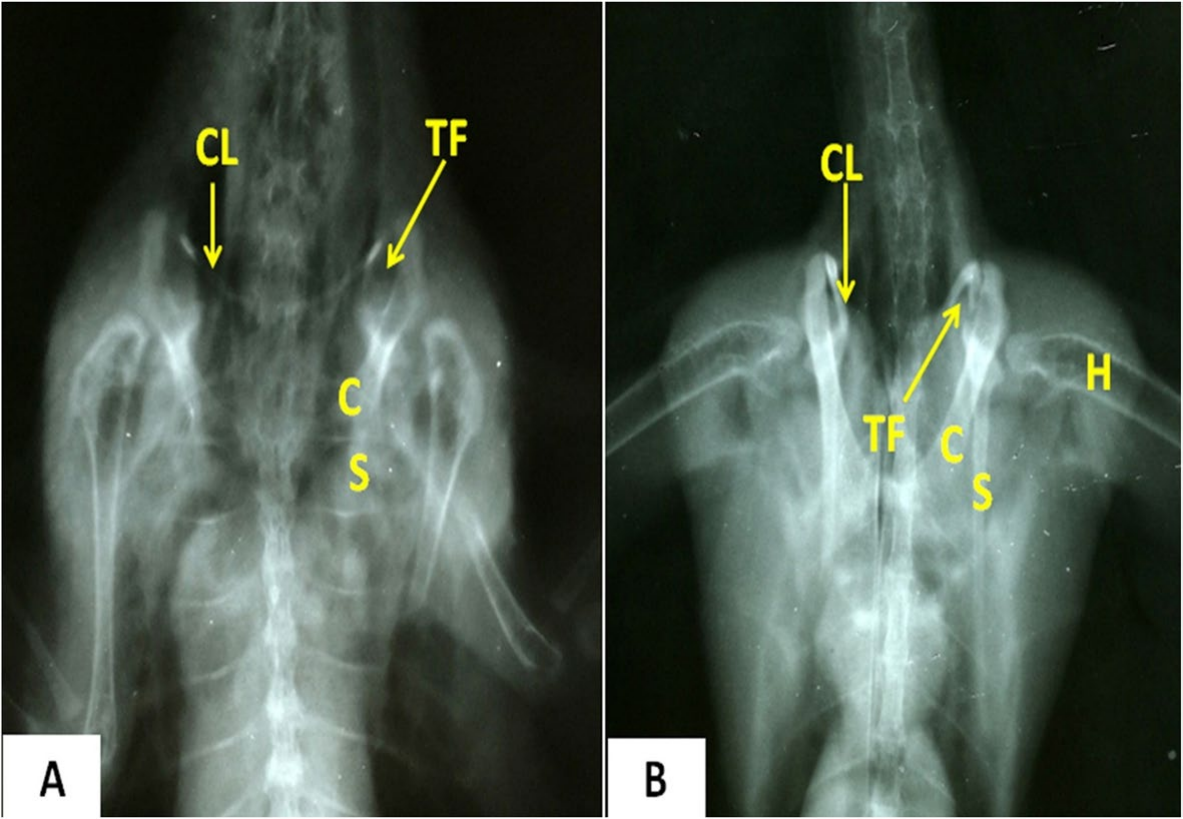
Radiographs of domestic ducks (**A**) and cattle egrets (**B**) showing bones forming the pectoral girdle and the triosseum foramen (ventrodorsal view). *S* Scapula, *C* Coracoid, *CL* Clavicle, *H* Humerus, *TF* Triosseum foramen

The statistical differences in the length of the sternum, scapula, coracoid, humerus, and clavicle in relation to the body length, as well as the width of triosseum foramen in domestic ducks compared to cattle egrets, are depicted in Fig. 1. The humerus of both species was considered one component of the wing skeleton (Figs. 2 and 3).

### Scapula

**Fig. 4:**
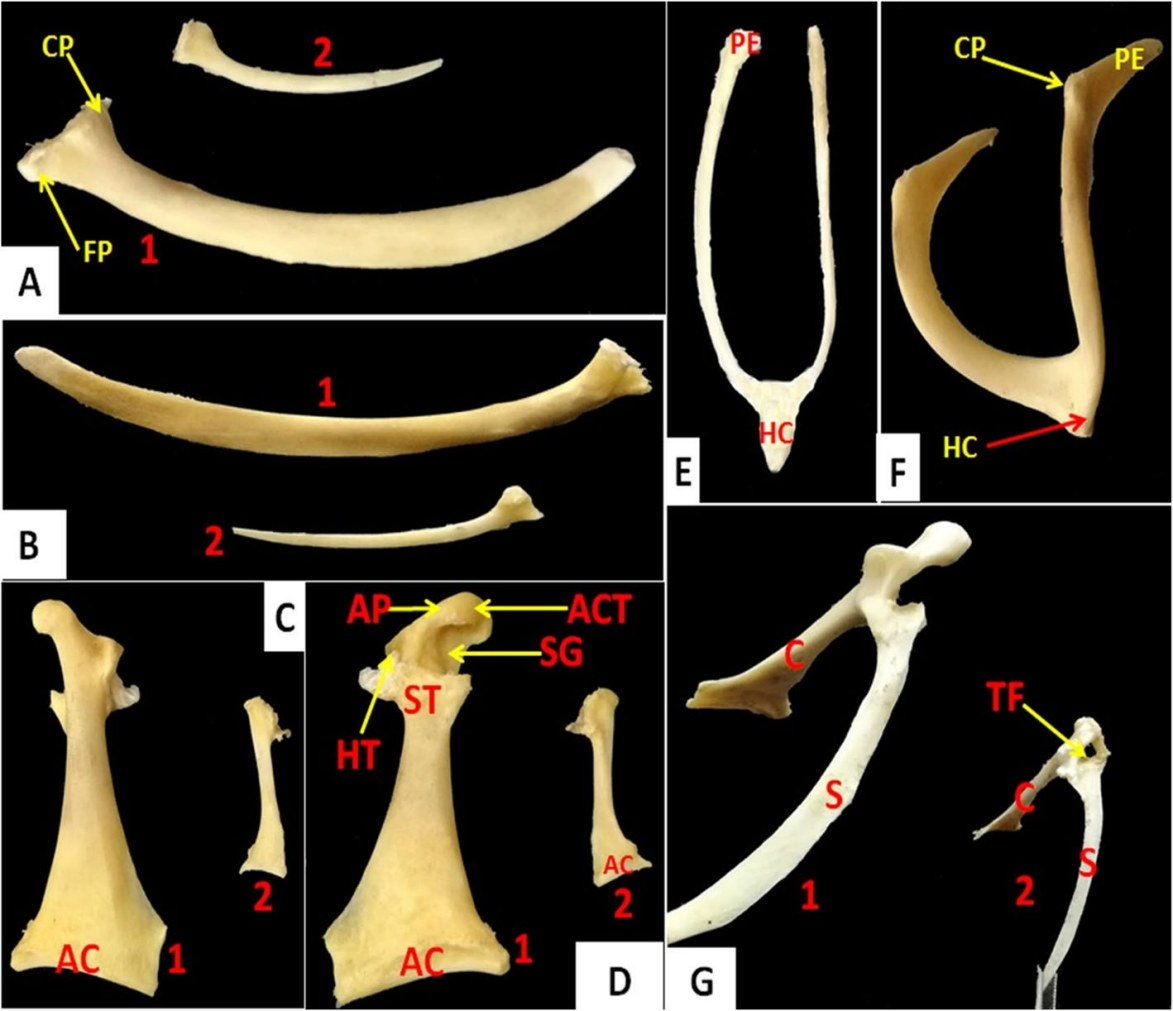
Photomacrographs of the scapula of domestic ducks (**A1**) and cattle egrets (**A2**) (ventral view). Photomacrographs of the scapula of domestic ducks (**B1**) and cattle egrets (**B2**) (dorsal view). Photomacrographs of coracoid of domestic duck (**C1**) and cattle egrets (**C2**) (dorsal view). Photomacrographs of coracoid of domestic ducks (**D1**) and cattle egrets (**D2**) (ventral view), Photomacrographs of frucula of cattle egrets (**E**) (cranial view) and domestic duck (**F**) (craniolateral view). Photomacrographs of the articulated scapula and coracoid of domestic duck (**G1**) and cattle egrets (**G2**) (dorsal view). *CP* Coracoidal process, *FP* Furcular process, *AC* Articular crest, *AP* Acromial process, *ACT* Acrocoracoid tuberosity, *HT* Humeral tuberosity, *SG* Supracoracoid groove, *ST* Scapular tuberosity, *PE* Proximal extremity, *Hc* Hypocladium, *S* Scapula, *C* Coracoid, *TF* Triosseum foramen

It was located dorsally at the lateral thoracic wall, parallel to the spine (Figs. 2 and 3). It appeared as a thick semi-curved elongated flat bone in the domestic duck (Fig. 2A), whereas in the cattle egret appeared as a thin rod-like bone (Fig. 2B). It consisted of cranial and caudal extremities and a shaft (Fig. 2C and D). The cranial extremity was wider than the caudal one. The caudal extremity was a thick rounded end in domestic ducks, whereas it was a thin pointed end in the cattle egret (Fig. 4A and B). The cranial extremity was found to form articulation with the clavicle, coracoid, and head of the humerus, forming furcular, coracoidal, and humeral processes (Fig. 4A and B). The shaft appeared thinner at the mid-shaft in domestic ducks than in cattle egrets, where the width and thickness were approximately equal along the shaft (Fig. 4A and B).

### Coracoid

It was thick and long in shape and extended ventrally from the shoulder joint to articulate with the sternum by a concave elongated facet on its caudal end **(**Figs. 2 and 3**)**. It consisted of two extremities and a shaft. The cranial extremity was narrower than the caudal one. The caudal extremity was spatula-shaped in domestic ducks whereas, in the cattle egret, it was extended laterally by a thin concave plate **(**Fig. 4C and D**)**. The cranial extremity was characterized by the presence of humeral, scapular, and acrocoracoid tuberosities, acromial process, and supracoracoid groove **(**Fig. 4C and D**)**. The acromial process is connected with the clavicle by connective tissue. The scapular tuberosity and coracoidal process of the scapula shaped the glenoid cavity for the articulation of the head of the humerus. Only in domestic ducks, the medial coracoscapular and cranial coracoclavicular ligaments were originally attached to the acrocoracoid tuberosity **(**Fig. 4G**)**.

### Clavicle

**Fig. 5:**
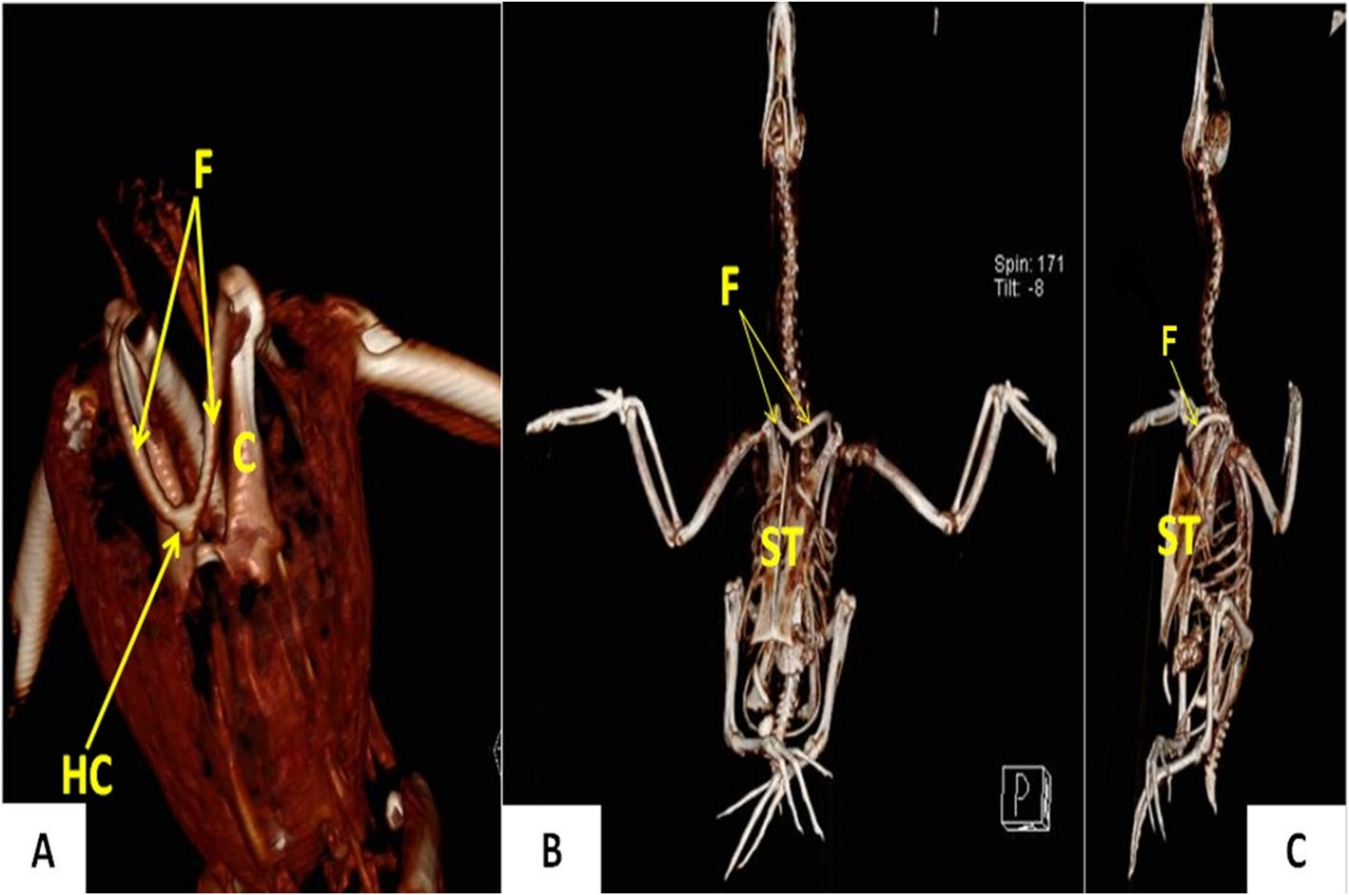
CT scan images of cattle egrets (**A**) and domestic ducks (**B**) (ventrodorsal view) and CT images of domestic ducks (**C**) (ventrolateral view) showing the connection between the sternum and the hypocladium of furcula by fibrous joint in cattle egrets and absence of this connection in domestic duck. *C* Coracoid, *F* Furcula, *ST* Sternum, *HC* Hypocladium

**Fig. 6:**
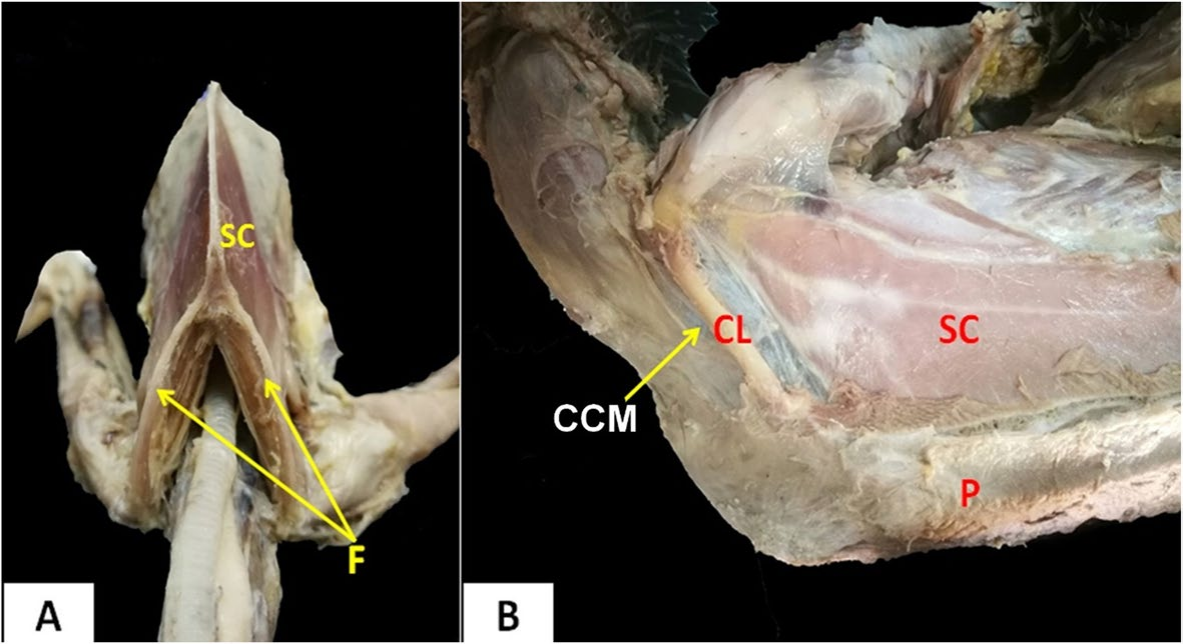
Photomacrographs of cattle egrets (**A**) (cranioventral view) and domestic ducks (**B**) (ventrolateral view). *F* Furcula, *CL* Clavicle, *CCM* Sterno-coraco-clavicular membrane, *SC* M. Supracoracoideus, *P* M. pectoralis

The clavicles were a curved bones (Figs. 2, 3 and 4). It appeared strap-like in cattle egrets and sickle-shaped in domestic ducks **(**Fig. 4E and F**)**. The pair of clavicle bones was found fused ventrally to form the furcula, where a small process called hypocladium was noted **(**Fig. 4E and F**)**. The hypocladium was connected to the sternum by a fibrous joint in cattle egrets (Fig. 5A), but this connection was absent in domestic ducks (Fig. 5B and C). We observed that the space between the clavicle and sternum was filled with a membrane (Sterno-coraco-clavicular membrane) that was fleshy-like in cattle egrets (Fig. 6A), whereas it was thin elastic fibrous tissue in domestic ducks (Fig. 6B).

### Humerus

**Fig. 7:**
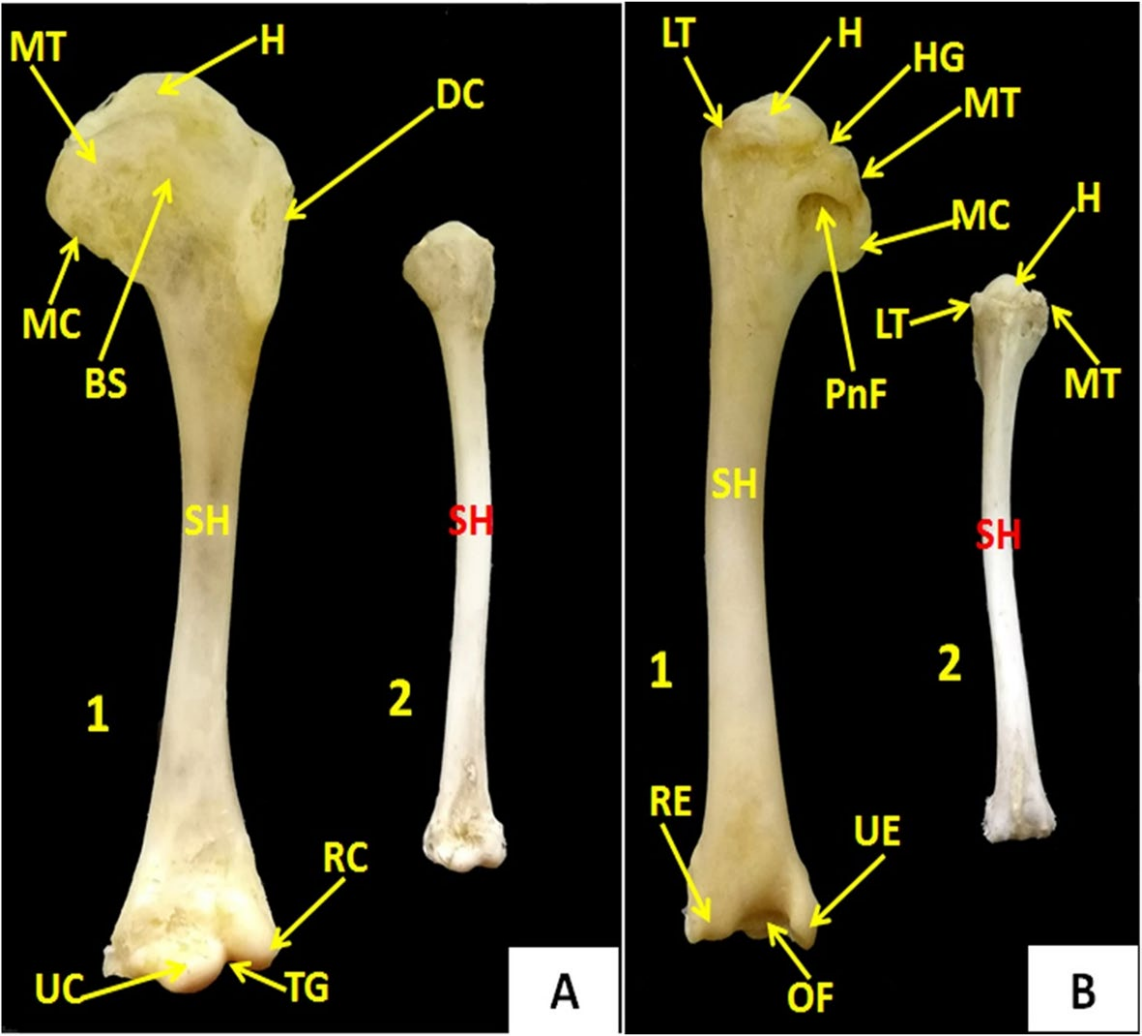
Photomacrographs of the humerus of domestic ducks (**A1**) and cattle egrets (**A2**) (cranial view), Photomacrographs of the humerus of domestic ducks (**B1**) and cattle egrets (**B2**) (caudal view). *SH* Shaft, *MT* Medial tuberosity, *LT* Lateral tuberosity, *H* Head, *DC* Deltoid crest, *MC* Medial crest, *BS* Bicipital surface, *RC* Radial condyle, *UC* Ulnar condyle, *RE* Radial epicondyle, *UE* Ulnar epicondyle, *TG* Trochlear groove, *HG* Head groove, *PnF* Pneumatic foramen, *OF* Olecranon fossa

It was a long bone formed by the proximal and distal epiphyses, and the diaphysis (Fig. 2**)**. The proximal epiphysis contained a large head that was ovoid in cattle egrets and oval in domestic ducks (Fig. 7A and B**)**. On either side of the head, there were medial and lateral tuberosities. The lateral tuberosity extended on the lateral surface forming the deltoid crest and the medial one formed the medial crest (Fig. 7A**)***.* Between the head and the medial tuberosity, the head groove was present. In cattle egrets, the latter groove continued distally on the medial surface of the humerus by the coracobrachial groove. The deep pneumatic fossa contained a large pneumatic foramen (Fig. 7B**)** that was located distally to the medial tuberosity. On the lateral surface of the proximal diaphysis, between the deltoid and medial crest, there was a bicipital surface to which the dorsal coracohumeral ligament was attached (Fig. 7A).

**Fig. 8:**
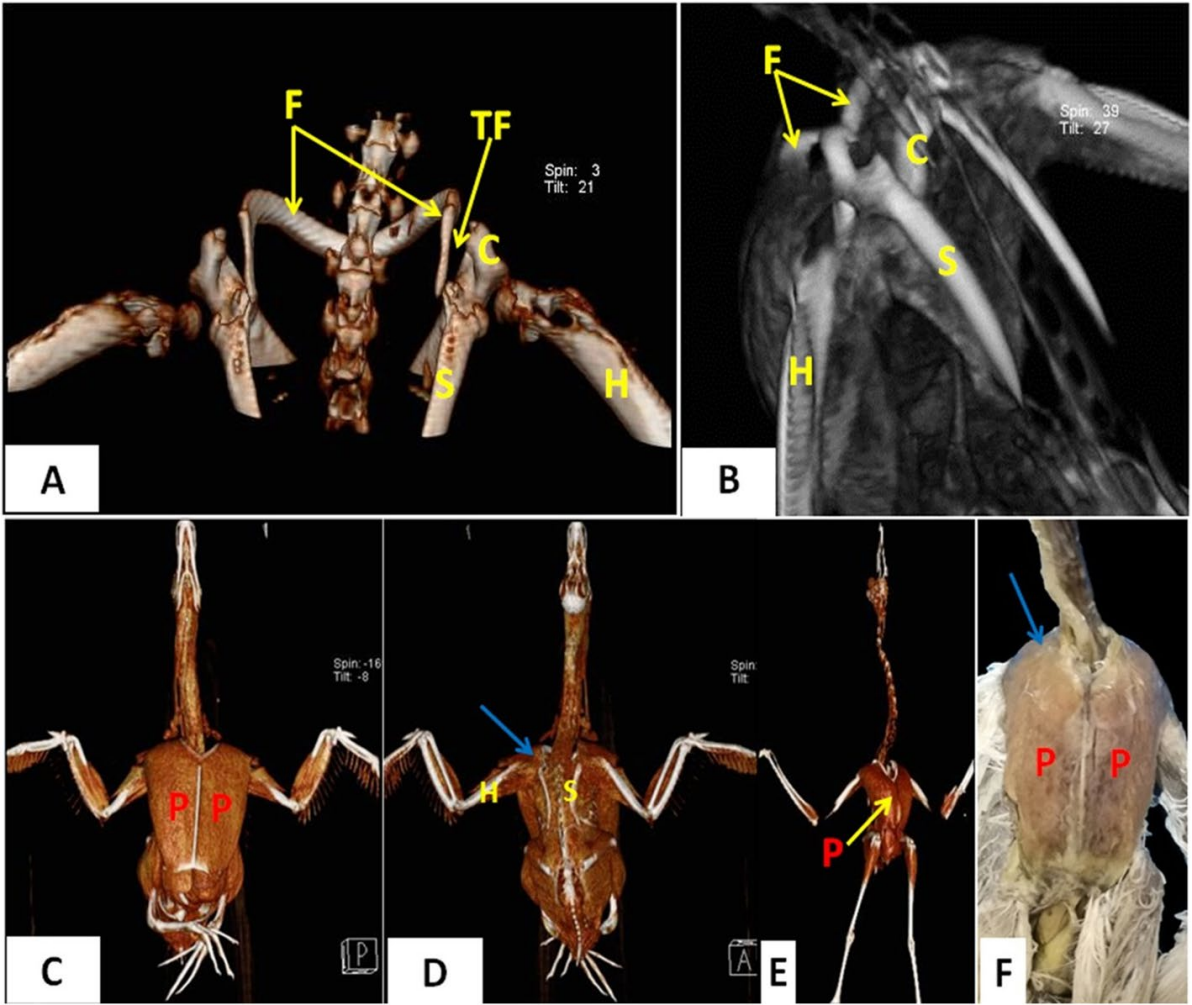
CT images of domestic ducks (**A**) (dorsal view) and cattle egrets (**B**) (dorsolateral view), CT images of domestic ducks (**C**) (ventral view) and (**D**) (dorsal view) and CT images of cattle egrets (**E**) and photomacrograph of cattle egrets (**F**) (ventral view). *S* Scapula, *C* Coracoid, *H* Humerus, *F* Furcula, *TF* Triosseum foramen, *P* M. pectoralis and blue arrow: showing the area of triosseum foramen that was wholly covered by muscle pectoralis

In the cattle egrets, the bones of the pectoral girdle (scapula, coracoids, and clavicle) articulated with each other forming triosseum foramen (Figs. 2, 3 and 8A and B). This region was completely covered by the external and internal muscle pectoralis (Fig. 8C. D, E and F). The latter muscle was the largest muscle that occupied the ventral surface of the sternum (Fig. 8C and E). It originated from the keel and body of the sternum and the lateral surface of the furcula and was inserted into the deltoid crest of the humerus. The M. pectoralis completely covered the M. supracoracoideus in the ventral aspect of the sternum (Fig. 6A and B).

**Fig. 9:**
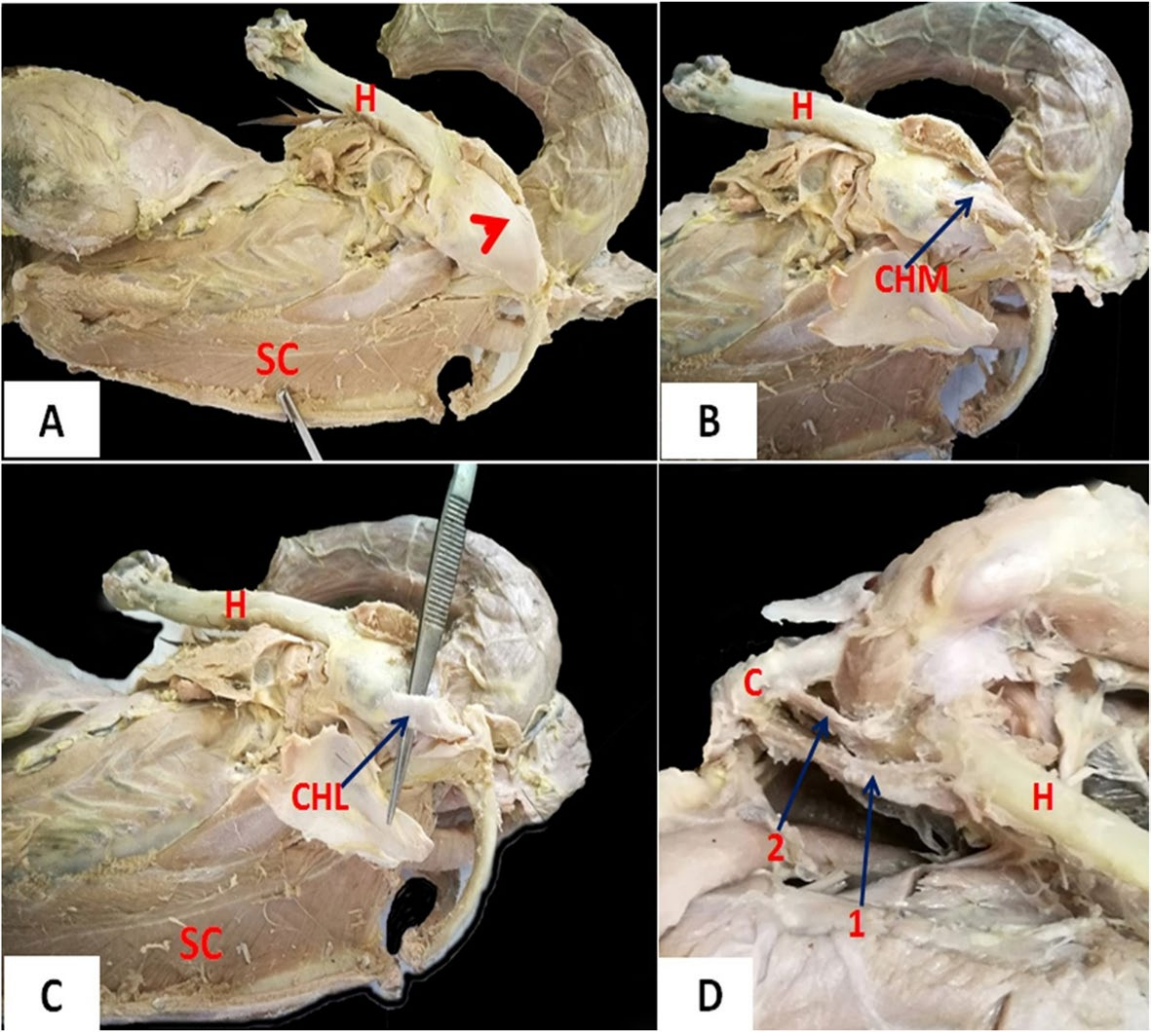
Photomacrographs of dissected domestic ducks (lateral view). *H* Humerus, *C* Coracoid, *SC* M. Supracoracoideus; arrowhead: strong joint capsule encircling the shoulder joint, *CHM* M. coracohumeralis dorsalis, *CHL* Coracohumeral ligaments. 1: Dorsal coracohumeral ligament, 2: Deep coracohumeral ligament

In domestic ducks, there was a thicker joint capsule encircling the shoulder joint (Fig. 9A) compared to cattle egrets. After the removal of the M. coracohumeralis dorsalis (originated from the distal extremity of the humerus and inserted near to acrocoracoid tuberosity of the coracoid) (Fig. 9B), the dorsal coracohumeral ligament and the underneath deep coracohumeral ligament were found. The dorsal coracohumeral ligament originated from the acrocoracoid tuberosity of the coracoid and inserted at the lateral tuberosity of the humerus (Fig. 9C). The deep coracohumeral ligament originated from the humeral tuberosity of the coracoid and ended at the medial tuberosity of the humerus (Fig. 9D).

**Fig. 10:**
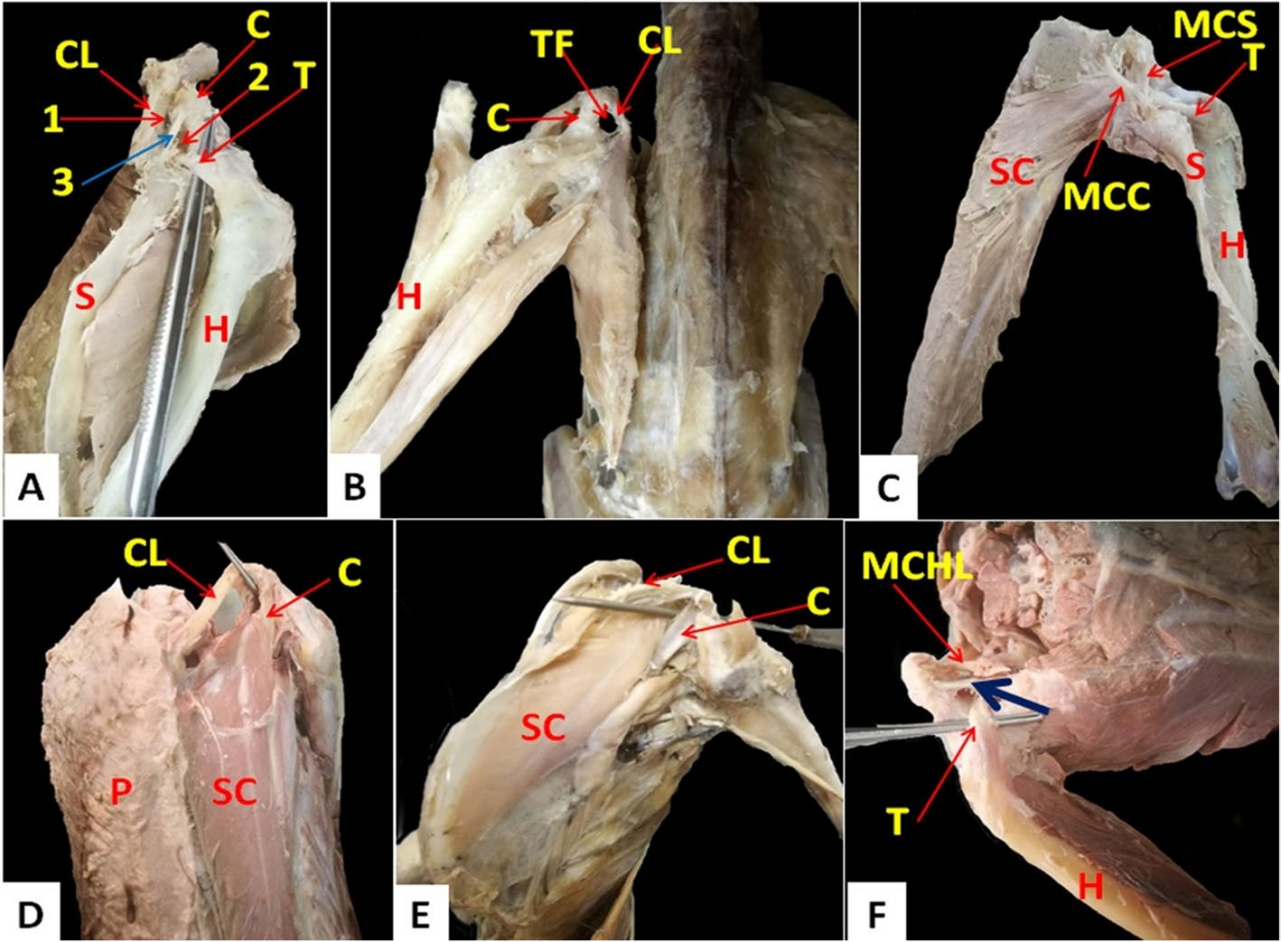
Photomacrographs of dissected domestic ducks (**A**) (dorsolateral view). Photomacrographs of dissected cattle egrets (**B**) (dorsal view). Photomacrographs of dissected domestic ducks (**C**) (dorsal view). Photomacrographs of dissected domestic ducks (**D**) (ventral view). Photomacrographs of dissected cattle egrets (**E**) (ventrolateral view). Photomacrographs of dissected domestic ducks (**F**) (dorsolateral view). *S* Scapula, *C* Coracoid, *CL* Clavicle, *H* Humerus, *TF* Triosseum foramen, *SC* M. Supracoracoideus, *P* M. pectoralis, *T* Tendon of the Supracoracoideus muscle. 1: Craniomedial part of triosseum foramen, 2: Caudolateral part of triosseum foramen, 3 (and blue arrow): Medial coracoclavicular and medial coracoscapular ligaments, *MCS* Medial coracoscapular ligament, *MCC* Medial coracoclavicular ligament, *MCHL* M. coracohumeralis lateralis

The triosseum foramen was an equilateral triangle in domestic ducks and was divided by two ligaments; medial coracoclavicular (originated from acromion process of coracoid then directed to be inserted at the proximal extremity of the clavicle) and medial coracoscapular ligament (originated from the acrocoracoid tuberosity of coracoid then directed to be inserted at coracoid process of the scapula) (Fig. 10A); the foramen bounded by the cranial coracoclavicular ligament (originated from the acrocoracoid tuberosity of coracoid then directed to be inserted at the proximal extremity of the clavicle). Whereas in cattle egrets, the triosseum foramen was a triangle with a cranially situated apex and was undivided. The clavicle cranially encircled the triosseum foramen (Fig. 10B).

In domestic ducks, there were three angles of the triosseum foramen: craniomedial, caudomedial, and caudolateral angles. The craniomedial angle was formed by the proximal extremity of the clavicle. The caudomedial was formed by the articular extremity of the scapula, while the caudolateral one was formed by the coracoid (Fig. 10A). The triosseum foramen was divided into two parts, craniomedial and caudolateral parts by medial coracoclavicular and medial coracoscapular ligaments (Fig. 10C). Through the caudolateral part, the tendon of insertion of the supracoracoid muscle was elongated fusiform (feather-like) and originated from the keel and body of the sternum and inserted into the head of the humerus (Fig. 10D). The M. coracohumeralis lateralis, which originated from the medial surface of the coracoid and furcular process of the scapula and was inserted in the deltoid crest), was found deeply close to the ligaments which divided the triosseum foramen (Fig. 10C and F).

In cattle egrets, the triosseum foramen was a well-differentiated triangle formed by the fusion of the proximal ends of the shoulder girdle bones. Also, the tendon of insertion of the M. supracoracoideus was found passing through it (Fig. 10B). The M. supracoracoideus appeared fusiform but darker in color and shorter in length than that of the domestic ducks and was inserted into the lateral tuberosity of the humerus (Fig. 10E). The caudomedial boundary of the triosseum foramen was formed by the proximal end of the scapula, while the craniomedial one was formed by the proximal end of the clavicle. In addition, the craniolateral boundary was formed by the proximal end of the coracoids (Fig. 10B and E).

### Histological findings

#### Pectoral muscles

**Fig. 11:**
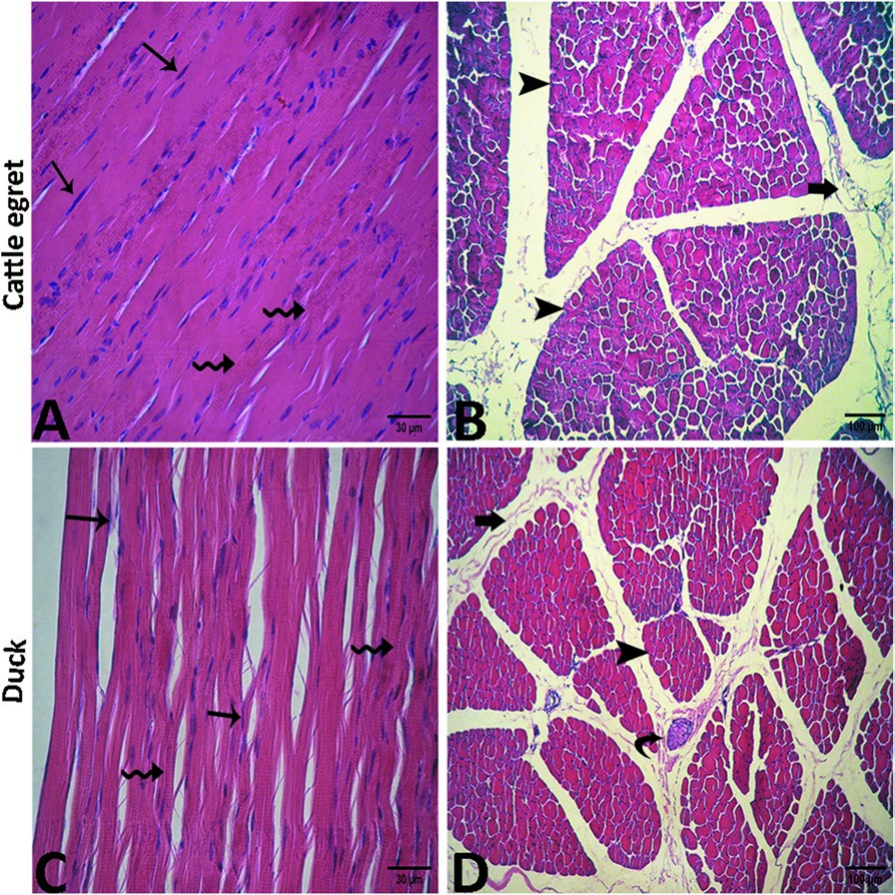
Photomicrograph of the pectoral muscle of the cattle egret (**A** and **B**) and the domestic duck (**C** and **D**) showing elongated nuclei "arrows", visible cross striations “zigzag arrows”, muscle bundles "arrowheads", perimysial connective tissue "thick arrows" and nerve fascicle "curved arrow"

In the cattle egret, the muscle bundles appeared thick and surrounded by dense perimysial connective tissue. The myofibers appeared long, cylindrical, compacted, and straight in shape as well as separated by narrow endomysial connective tissue. Hyper acidophilic cytoplasm was evident by the presence of evident cross striations. An extensive number of elongated peripherally located nuclei were observed (Fig. 11A). The cross section of the pectoral muscle showed perimysial connective tissue spaces among the bundles and some muscle fibers displayed hyper acidophilic cytoplasm with narrow endomysial connective tissue spaces in between **(**Fig. 11B**)**. In the domestic duck, the perimysium separating muscle bundles consisted of less dense connective tissue. The myofibers were long, cylindrical, and wavy in shape with acidophilic cytoplasm. Multiple elongated peripherally located nuclei were noted. The myofibers were loose and contained numerous endomysial connective tissue spaces **(**Fig. 11C). Cross section of this muscle showed several muscle bundles surrounded by the perimysium, which consisted of less dense connective tissue containing blood vessels and nerve fascicles **(**Fig. 11D**)**.

#### Supracoracoideus muscles

**Fig. 12:**
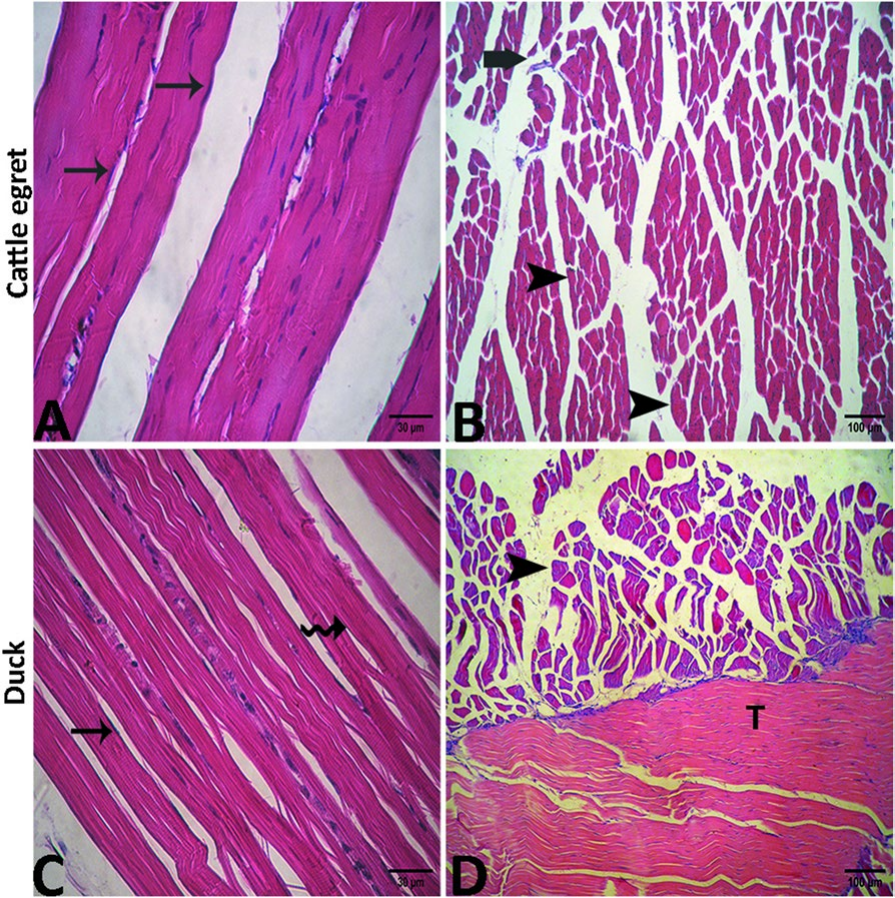
Photomicrograph of the supracoracoideus muscle of the cattle egret (**A** and **B**) and the domestic ducks (**C** and **D**) showing elongated nuclei “arrows”, muscle bundles “arrowheads”, perimysial connective tissue “thick arrow”, visible cross striations “zigzag arrows”. Also, note the tendinous part of the muscle "T"

In the cattle egret, the muscle bundles appeared thin and elongated in shape with acidophilic cytoplasm. There was a little perimysial connective tissue between muscle bundles. The myofibers were highly compacted with narrow endomysial space and peripherally located nuclei which appeared fewer in number than the pectoral muscle (Fig. 12A). In the cross-section, there was little connective tissue between muscle bundles. The muscle fibers were highly compacted to each other with narrow endomysial space **(**Fig. 12B**)**. In domestic ducks, the muscle bundles appeared cylindrical in shape with thinner wavy myofibers. (Fig. 12C). In cross-section, the myofibers were slightly irregular or ovoid in shape. Also, it appeared thinner, and with wider endomysial spaces compared with that of cattle egret. Also, the tendinous part of the muscle consisted of dense regular collagenous connective tissue and appeared wavy in appearance and pale in color with oval nuclei (Fig. 12D). Histomorphometry revealed a highly significant difference (*p* < 0.001) between cattle egrets and ducks in the thickness and area % of connective tissues of pectoral and supracoracoideus muscle bundles (Table 3). Additionally, the number of nuclei in the pectoral and supracoracoideus muscle was significantly higher (*p* > 0.05) in cattle egrets compared with that in domestic ducks (Table 4).

**Table 3:** Histomorphometric measurements representing the mean ± standard error of thickness of muscle bundles of pectoral and supracoracoideus muscles of both cattle egret and domestic duck and area % of connective tissue of pectoral and supracoracoideus muscles of both cattle egret and duck

Parameters	Cattle egret	Domestic duck	p-value
Thickness of muscle bundles (um) of the pectoral muscle	329.8 ± 9.33	242.0 ± 12.33	< 0.0001
Thickness of muscle bundles (um) of supracoracoideus muscle	195.8 ± 12.41	162.5 ± 12.8	= 0.0013
Area % of connective tissue in the pectoral muscle	1.90 ± 0.26	0.67 ± 0.12	< 0.0001
Area % of connective tissue in supracoracoideus muscle	0.5 ± 0.10	0.19 ± 0.10	= 0.0005

**Table 4:** Histomorphometric measurements representing the mean ± standard error of the number of nuclei in pectoral muscle and supracoracoideus muscle of both cattle egret and domestic duck

Parameters	Cattle Egret	Domestic duck	p-value
Number of nuclei per defined area (400 um^2^) in pectoral muscle	5.0 ± 1.25	4.0 ± 1.25	< 0.05
Number of nuclei per defined area (400 um^2^) in supracoracoideus muscle	3.0 ± 0.5	2.0 ± 1.25	< 0.05

### Immunohistochemical findings

#### Pectoral muscles

**Fig. 13:**
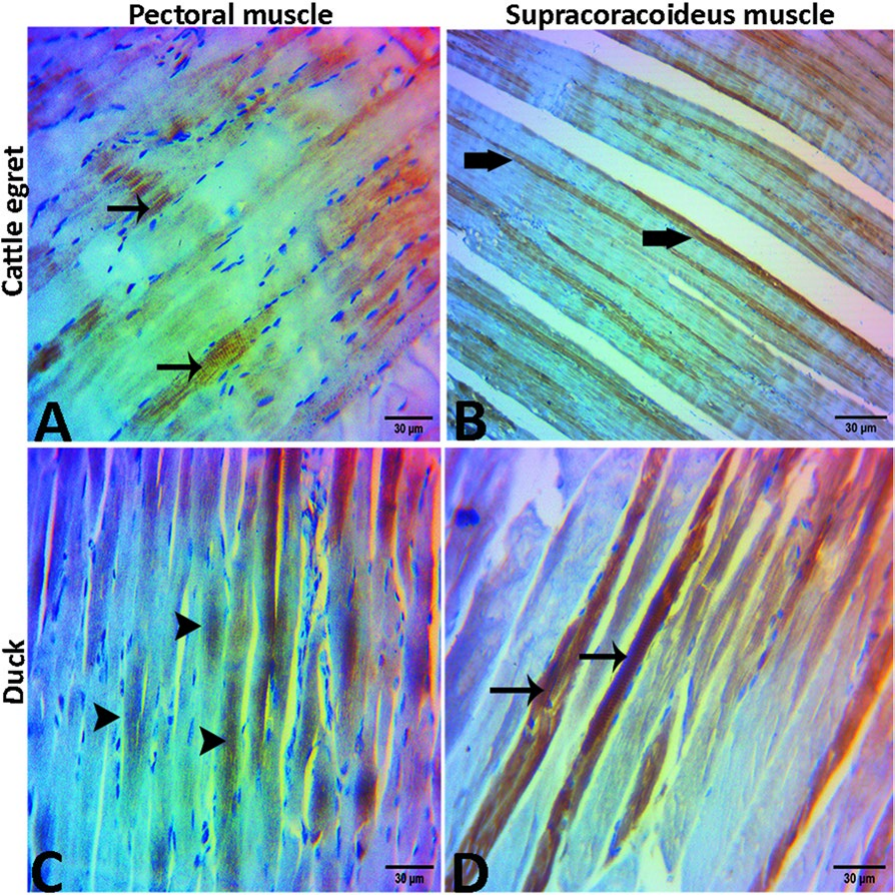
Photomicrograph of immunohistochemically stained section against myoglobin antibody showing (**A**) cattle egret’s pectoral muscle was positively reacted particularly at cross striations "arrows". (**B**) Supracoracoideus muscle of cattle egret showed homogenous densely stained color "thick arrows". (**C**) The pectoral muscle of the domestic duck reacted positively in the vicinity of the sarcoplasm "arrowheads". (**D**) Supracoracoideus muscle fibers of domestic ducks were immuno-stained by myoglobin antibody, particularly the cross striations "arrows"

In the cattle egret, the pectoral muscles exhibited a high myoglobin expression in the cytoplasm of the muscle fibers and were detected mainly in the Z-line and cross-striations (Fig. 13A). In the pectoral muscle of the domestic duck, the positive expression of the myoglobin was mainly detected in the vicinity of the sarcoplasm, especially beneath sarcolemma or around the nuclei. The intensity of the reaction appeared identical and evenly distributed among the different fibers (Fig. 13C).

#### Supracoracoideus muscle

In cattle egret, expression of myoglobin in the supracoracoideus muscle was high and homogenously distributed along the myofibers. Of note, the striation of myofibers showed more myoglobin expression in the pectoral muscle than in the supracoracoideus muscle (Fig. 13B). In the domestic duck, the supracoracoideus muscle fibers were positively stained for myoglobin, particularly at the cross striations. As noted, the staining appeared strong in some myofibers and weak in others (Fig. 13D).

## Discussion

The present study reported that the pectoral girdle of both species consisted of three pairs of bones: scapula, coracoid, and fused clavicles. The humerus of both species was considered one component of the wing skeleton. These findings came in the same line as reported in domestic pigeons [9], domestic birds [10], domestic fowl [25], and crows and owls [26]. The articulation of the pectoral girdle bones leads to the formation of triosseum foramen as observed in this study and in cattle egrets [27].

In the current study, there was a clear difference between the scapula of domestic ducks and cattle egrets. Its shape was a thick curved plate in the domestic ducks while in the cattle egrets was a thin rod-like plate. In pigeons, crows, and owls, the scapula was a blade-like structure and slightly curved from the cranial to the caudal direction [26]. While in domestic pigeons [9] the scapula appeared sword in shape with much-reduced acromion, much smaller in relation to body size. In pigeon Hawk and Kite, there was pneumatic foramen just behind the coracoid process of the scapula of the pigeon hawk. However, the scapula of the kite was characterized by the presence of subacromial pneumatic foramen [28]. The medial process of the scapula of the crow was perforated by a pneumatic foramen which was absent in both owl and pigeon. The presence of an additional intermediate process and pneumatic foramina could act as an identification mark for the scapula of crows [26]. These pneumatic foramina were absent in both examined species in this study.

Concerning the coracoid bone of domestic ducks and cattle egrets, it extends ventrally from the shoulder joint to articulate with the sternum and is characterized by its cranial extremity, which was narrower than the caudal one. The Coracoid was directed downwards and backward to articulate the sternum distally. The important role of the coracoid in holding the wings away from the sternum during the flight was confirmed [29]. The proximal extremity of the coracoid was hook-like and articulated with the proximal extremity of the scapula and clavicle to form the foramen triosseum [9] in domestic pigeons. The strong development of supracoracoideus and sternocoracoideus muscles and the more strongly developed coracoid of kite improved their adaptation feature to catch their prey during flight [28]. The coracoid bones are pillars that join the avian shoulder to the thorax and convey forces to the sternum to participate in the elevation of the wing above the level of the vertebral column through its supracoracoideus tendon [30]. Furthermore, at their joint with the sternum, the coracoid bones rotate and slide allowing a spring-action of the clavicles with each wing beat [30]. The changes in the coracosternal joint (fracture of the coracoid, luxation, arthritis) convince pain with every wing beat and may lead to drooped wings [31]. The space enclosed between the procoracoid and acrocoracoid process is the major contributor to the foramen triosseum [28].

In the present study, furcula was formed by a fusion of sickle-shaped clavicles in domestic ducks while it was strap-like in cattle egrets. The clavicles were two in number – right and left which were fused distally to form a single bony structure called furcula in pigeons, crows, and owls [26]. Furcula had different shapes according to the species; domestic pigeons had a broad 'U' shaped furculum with rudimentary hypocleideum [9]. It was U shaped in crows [32] and pariah kite [33] while in fowl the furculum appeared a wide 'V' shape [25]. Also, in owls and pigeons, it was ‘V-shaped [26]. In our study, the furcula of domestic ducks had a wide V shape, while cattle egrets had an elongated narrow U shape. The variation in the morphology of furcula was correlated with the flight requirements of different birds [26]. Another report opined that the furcula, generally absent in non-flying birds, may act as an essential component during flight [34]. At the site of the fusion of the two clavicles, there was hypocleideum connected to the sternum by a fibrous joint in cattle egrets, but this connection was absent in domestic ducks. The hypocleideum was absent in pigeons and owls [26]. The hypocleideum contacted the sternum either directly or via a ligamentous attachment [8]. In fowl, the hypocleideum was a thin oval plate [25], while in crow it was in the form of a flattened 'S-shaped sagittal plate [32]. The hypocledium fused to the ventral part of the cranial border of the sternal crest permanently [35] in spot-billed pelican and [36, 37] in pelicans and frigate birds. The latter authors suggested that the fusion created rigidity in the pectoral girdle, which assisted in soaring with moderately small breast muscles. Although, in domestic birds, they were separated, the hypocledium was attached to the sternal crest by a membrane [38]. In the current work, we observed that there was a membrane filled the space between the sternum, coracoid, and clavicle (Sterno-coraco-clavicular membrane) that was thin elastic fibrous tissue in domestic ducks, whereas it was fleshy-like in cattle egrets. The presence of the Sterno-coraco-clavicular membrane stretched as an unpaired structure between the sternum, furcula, and coracoids was confirmed by [39] in *Psittacus erithacus*.

The humerus of domestic ducks and cattle egrets was a long bone with a large head that was ovoid in cattle egrets, as also noticed in pariah kite [40] and in cattle egrets and oval in domestic ducks. While the humerus of an Emu lacked a distinct head [41]. We observed in both examined species large pneumatic foramen inside the deep pneumatic fossa, which was located distally to the medial tuberosity. Similar results were noticed in long-legged buzzards [42] and in cattle egrets [43]. The weak flying ability of red-wattled lapwing as compared to pigeons and crows was due to lacking pneumatic foramen in the sternum and shoulder girdle, the presence of a relatively short coracoid, and the absence of hypocledium [34]. The humerus, ulna, tibiotarsus, and tarsometatarsus of cattle egrets were long [44] and regulated flying ability according to the weight-strength-rule [45].

In the current study, the triosseum foramen was an equilateral triangle in domestic ducks and was divided into two parts by two ligaments: medial coracoclavicular and medial coracoscapular ligament. The foramen bounded by the cranial coracoclavicular ligament. Whereas in cattle egrets, the triosseum foramen was a triangle with a cranially situated apex and was undivided. The clavicle cranially encircled the triosseum foramen. In domestic ducks, there were three angles of the triosseum foramen, craniomedial, caudomedial, and caudolateral angles, while in cattle egrets these angles were caudomedial, craniomedial, and craniolateral. While John et al.,  [34] stated that, the anterio-medial boundary of this foramen was formed by the proximal end of the clavicle and the anterio-lateral boundary was formed by the proximal end of the coracoid. The posterior and a part of the medial boundary were formed by the proximal end of the scapula. The presence of a hook-like proximal extremity of the coracoid facilitates the supracoracoideus muscle for better support and thus aids in flight.

Our investigations about M. pectoralis revealed that it was the largest muscle that occupied the ventral surface of the sternum that completely covered M. *supracoracoideus* in the same line with Razmadze et al. [39] in *Psittacus erithacus*. The latter author added that M. *pectoralis* was formed of three parts: the largest pars thoracicus and two additional ones: *pars propatagialis longus* and *pars propatagialis brevis.* In examined domestic ducks, M. s*upracoracoideus* appeared elongated fusiform (feather-like) and extended along the sternum up to its caudal margin. On the contrary, M. *supracoracoideus* of cattle egrets appeared darker in color than domestic ducks and occupied the cranial part of the sternum. The extension of M. *supracoracoideus* along the sternum differed from one species to another. In *Psittacus erithacus*, it extended along the full length of the sternum right up to its caudal edge [39], while in falcons, it appeared small and found only on the cranial half of the sternum [46]. Concerning the tendon of insertion of M. *supracoracoideus* in domestic ducks, it passed through the caudolateral part of the triosseum foramen and inserted into the head of the humerus, while in cattle egrets it found inserted into the lateral tuberosity of the humerus. Moreover, Razmadze et al., [39] stated that, after passing through the triosseum foramen, the tendon was inserted on the dorsocaudal surface of the humerus just distal to the tuberculum dorsal.

However, more literature was needed on the morphometry of the shoulder girdle of domestic ducks and cattle egrets. Hence, the present study was carried out in these species. Regarding the morphometric analysis of different bones under examination, by comparison, the length of the examined bones and the total body length of the studied species, we observed that the relative length of the sternum to the total body length was 22.9% ± 1.52 in domestic ducks while in cattle egrets, it was 14% ± 1.25. The relative length of the humerus represented 22.87% ± 1.43 and 21.4% ± 1.35 to the total body length in domestic ducks and cattle egrets, respectively. In domestic ducks, the relative length of the scapula, coracoid, and clavicle to the total body length was 18.4% ± 1.51, 12.3% ± 0.82, and 12.3 ± 0.67 respectively. While in cattle egrets, the relative length of the scapula, coracoid, and clavicle to the total body length was 11.4% ± 1.17, 8.7% ± 0.48, and 10.1% ± 0.74, respectively. So, the relative length of all examined bones to the total body length in domestic ducks was significantly longer (*p* < 0.05) than in cattle egrets. The length of the scapula was 10.93 cm ± 0.49 in domestic ducks, while in cattle egrets, it was 4.71 cm ± 0.45, similarly to that obtained by Parvez et al., [9] in domestic pigeons. The latter author reported that the length and maximum width of the scapula were 4.23 cm and 0.56 cm, respectively. The Coracoid length was 3.53 cm, and the width of the distal extremity (1.45 cm) was about twice that of the proximal one (0.70 cm). The length of the coracoid in geese was 1.50 cm which is almost similar to fowl, but the width of extremities was larger compared to fowl [47]. The length of coracoid bone was maximum in crows (4.6 cm) followed by owls (3.8 cm) and least in pigeons (3.6 cm) [26]. The maximum length of the right and left humerus of cattle egrets was 90.78 ± 0.94 mm for the right humerus and 90.72 ± 0.89 mm for the left humerus [44]. The maximum length of the humerus of the long-legged buzzard was 10.5 cm [42]. While in our study, the maximum length of the humerus was 14 cm in domestic ducks and 9 cm in cattle egrets.

The muscle pectoralis, as the primary flight muscle in birds, has been evaluated histologically in some avian species [11] and for its mechanics in the red-tailed hawk and barred owl [48]. In this study, we found that the muscle bundles (fascicles) and myofibers of both the muscles pectoralis and supracoracoideus showed histologically more nuclei as well as numerous and narrower perimysial and endomysial spaces in cattle egret than those in domestic duck. For another comparison, each of these muscles in the pigeons showed microscopically abundant muscle fibers and nuclei in addition to wider perimysial spaces compared with chickens [11].

Computer-generated imaging has been recently introduced for avian examination. Of these studies, computer-generated radiology was used to assess the trabeculae of tibiotarsal bones in domestic ducks [20]. Moreover, in domestic ducks, the brain and skull are demonstrated using conventional radiography and CT [49, 50] as well as MRI [49]. Herein, the bones of the pectoral girdle and the triosseum foramen were clearly visualized and identified using conventional radiographic and 3D CT imaging. Also, computed tomography was used to evaluate the morphological changes of duck (Anas platyrhynchos) tibiotarsal bones [51].

In broiler chickens, myofibers of muscle pectoralis contain perimysium consisting of dense connective tissue that collects muscle fibers in bundles (fascicles) [52]. In our work, we found that the pectoral muscle bundles of domestic ducks were surrounded by perimysium and endomysium which consisted of less dense connective tissue relative to cattle egrets. These connective tissue-containing spaces are essential for the viability of muscle fibers by providing a space between muscle bundles and muscle fibers and structural support, thus maintaining the elasticity, or stretching of muscles. Moreover, these spaces contain capillaries that are essential for the activity of adult muscle cells and satellite cells and for the removal of respiratory by-products such as lactic acid [53].

This work showed that the pectoral muscles of cattle egrets have thick muscle bundles with abundant capillaries. The same results have been observed in domestic fowl but with few capillaries. The muscle fascicles and fibers are also thick, and their diameter is greater in hummingbirds that are structurally adapted to fly helping them move quickly between flowers to meet metabolic requirements [54]. Whereas, in the pigeon and bananaquit, it has been observed that the pectoral muscle has very thin muscle bundles with large abundant blood capillaries [55].

Our findings revealed that the muscular fibers of the cattle egret were long and cylindrical in shape and contained an acidic cytoplasm and more elongated peripheral nuclei than in domestic ducks. Other studies documented this finding and added that polynuclear cells are formed through the fusion of several muscle blasts during evolution [13, 14]. We also found that the pectoral muscles showed some fiber with a hyper acidophilic cytoplasm which was also noted in flying birds such as a pigeon, however unlike chickens [14].

Our findings revealed that the pectoral muscle fibers of domestic ducks were long, cylindrical, and loosely connected to each other. One of the most characteristic results was the number of nuclei that were lower in domestic ducks than in cattle egrets. Consistency, similar results have been reported in both broiler and domestic chickens indicating fewer nuclei were noted than that in pigeons [56].

It has been reported that domestication changes numerous neurological and physiological features of domestic ducks compared to their wild ancestors. Changes in the body size, bone morphology, and breast muscles compared to ancestral species were the main alterations. Domestic ducks also showed a loss of capability of flight and migration, decreased brain size, and increased body size [57]. Thus, as we found herein from morphological and histological findings, domestication may affect the physiological, anatomical, and histological structure of the components of the duck's locomotor system.

The supracoracoideus is considered the second largest wing muscle and has an antagonizing effect on the muscle pectoralis [8, 58]. In our study, the muscle supracoracoideus muscles of cattle egrets had red and thinner elongated muscle bundles with numerous nuclei, unlike domestic ducks whose muscle bundles appeared pale and thick cylindrical with fewer nuclei. In previous studies, this muscle in pigeons appeared darker in color compared to broilers and domestic chickens, and this explains why pigeons can fly higher than broilers and domestic chickens. Redness of the primary flight muscles is due to an increased number of muscle fibers, nuclei, and mitochondria [11, 13].

As noticed herein, both flight muscles of cattle egrets had a greater thickness of muscle bundles with narrower connective tissue spaces supporting their flying behavior during the flight compared with domestic ducks which had a lower thickness of muscle bundles, and the areas of peripheral connective tissue were wider and contained more connective tissue than those of cattle egret. The same results have been obtained in hummingbirds as flying birds, and in bananaquit as nonflying birds [54].

It has been shown that immunostaining of myoglobin is limited to the I-band, Z-line, mitochondrial outer membrane, and inner membrane of the sarcoplasmic reticulum [59]. We observed the same results in cattle egrets where the expression of myoglobin appeared mainly in the Z-line and the cross-striations. Overall, we should also mention that the expression was more pronounced in the pectoral muscle than in the supracoracoideus muscle. In conjunction with these data, the pectoral muscle is the largest and most energy-consuming organ in flying birds [60]. It is well-known that a higher myoglobin content enables muscle fibers to contract slowly and for longer periods allowing an increase in oxygen reserves. In domestic ducks, we found that pectoral and supracoracoideus muscle cells reacted positively to the myoglobin antibody which was mainly expressed in the vicinity of the sarcoplasm beneath sarcolemma.

## Conclusions

Despite several studies have evaluated the avian flight muscles, to the best of the authors’ knowledge, there is no information regarding the comparative evaluation of the flight muscles and bones in domestic ducks and cattle egrets. Domestic ducks lose their ability to fly depending on many factors, including their size, weight, wing structure, condition, and environment. Thus, this work sheds new light on the adaptive morphological characteristics of the pectoral girdle (bones and muscles) that suit the lifestyle of domestic ducks and cattle egrets. This study suggests that the bones and muscles of the pectoral girdle generally show specific morphological and structural changes reflective of the loss of basic requirements associated with flight behavior in domestic ducks due to domestication effects compared to cattle egrets.

## Data Availability

The datasets used and/or analyzed during the current study are available from the corresponding author (AA-I) upon a reasonable request.
